# *Hoxb4* Overexpression in CD4 Memory Phenotype T Cells Increases the Central Memory Population upon Homeostatic Proliferation

**DOI:** 10.1371/journal.pone.0081573

**Published:** 2013-12-06

**Authors:** Héloïse Frison, Gloria Giono, Paméla Thébault, Marilaine Fournier, Nathalie Labrecque, Janet J. Bijl

**Affiliations:** 1 Hospital Maisonneuve-Rosemont Research Center, Montreal, Quebec, Canada; 2 Department of Microbiology, Infectiology and Immunology, University of Montreal, Montreal, Quebec, Canada; 3 Department of Medicine, University of Montreal, Montreal, Quebec, Canada; University of Alberta, Canada

## Abstract

Memory T cell populations allow a rapid immune response to pathogens that have been previously encountered and thus form the basis of success in vaccinations. However, the molecular pathways underlying the development and maintenance of these cells are only starting to be unveiled. Memory T cells have the capacity to self renew as do hematopoietic stem cells, and overlapping gene expression profiles suggested that these cells might use the same self-renewal pathways. The transcription factor *Hoxb4* has been shown to promote self-renewal divisions of hematopoietic stem cells resulting in an expansion of these cells. In this study we investigated whether overexpression of *Hoxb4* could provide an advantage to CD4 memory phenotype T cells in engrafting the niche of T cell deficient mice following adoptive transfer. Competitive transplantation experiments demonstrated that CD4 memory phenotype T cells derived from mice transgenic for *Hoxb4* contributed overall less to the repopulation of the lymphoid organs than wild type CD4 memory phenotype T cells after two months. These proportions were relatively maintained following serial transplantation in secondary and tertiary mice. Interestingly, a significantly higher percentage of the *Hoxb4* CD4 memory phenotype T cell population expressed the CD62L and Ly6C surface markers, characteristic for central memory T cells, after homeostatic proliferation. Thus *Hoxb4* favours the maintenance and increase of the CD4 central memory phenotype T cell population. These cells are more stem cell like and might eventually lead to an advantage of *Hoxb4* T cells after subjecting the cells to additional rounds of proliferation.

## Introduction

Memory T cells develop from a small subset of effector T cells following a primary immune response. While effector T cells undergo apoptosis, memory T cells survive and provide the host an immunological memory allowing a faster and more effective immune response against previously encountered pathogens. Memory T cells are long-lived cells and their survival after antigen clearance depends on the homeostatic cytokines interleukin (IL)-7 and IL-15 [1]–[5]. Memory T cells persist by undergoing a slow turn-over, also referred to as basal homeostatic proliferation, with a frequency of one division in 2–3 weeks [3]. However, upon transfer into a lymphopenic host, memory T cells divide rapidly due to an increased availability of IL-7 and IL-15 [1]–[6], a phenomenon indicated as acute homeostatic proliferation. Knock-out mouse models for IL-15, IL-7 and IL-7Rα demonstrated that CD4 and CD8 memory T cells have a differential dependence for these cytokines. In the absence of IL-15 the basal homeostatic proliferation of CD8, but not CD4 memory T cells was severely reduced [1], [7], [8], while CD4 memory T cells fail to persist upon transfer into IL-7 deficient hosts [9]. However, acute homeostatic proliferation of both CD4 and CD8 memory T cells can be induced by either IL-15 or IL-7Rα signalling [1], [3], [10]. In addition to IL-7 and IL-15, which are the key factors for the survival and homeostatic proliferation of memory T cells, other cytokines have been shown to boost their homeostatic proliferation, such as IL-2 and interferon-1 (IFN-I) [11]–[13]. Despite their independence for T cell receptor (TCR) signalling to survive, experiments using knock-out mice showed that antigen specific CD4 memory T cells had reduced responses to antigen re-encounter in the absence of major histocompatibility complex (MHC) II [14]. Moreover, the presence of MHC II signals influenced the homeostatic expansion capacity of memory T cells under lymphopenic conditions, but this appeared to be independent on the avidity for MHC II, in contrast to naïve T cells [15]. This suggests that regulatory mechanisms governing memory homeostasis are different from naïve T cell homeostasis, which is important to maintain optimal diversity of the memory pool.

In addition to antigen-experienced memory T cells (true memory) a population of immunophenotypically identical memory cells exists that arise from interactions of the T cell receptor with endogenously expressed antigens [16] and are also referred to as memory phenotype (MP) T cells. Similarly to antigen-experienced memory cells, MP T cells are proliferating in response to lymphopenia and at least for CD8 it has been shown that they provide protection against antigen [3], [17], [18]. The requirements for homeostatic proliferation of MP T cells are slightly different than for true memory T cells. In addition to IL-15 and IL-7 they are dependent on MHCII [3], [19], [20], likely to avoid competition for signals provided in the niche.

Despite our increasing knowledge on the required signals, the molecular pathways behind homeostatic proliferation are still elusive. Some transcription factors have been shown to induce the expression of IL-7R or CD122 and thus allowing their permissive state to homeostatic survival and proliferation signals. For example, Foxo1 and GABPα promote IL-7R expression in T cells [21], [22]. In contrast, transcription factor Gfi-1 downregulates IL-7R expression by inhibiting GABPα following TCR signalling or cytokine stimulation [23]. On the other hand, transcription factors T-bet and Eomes are found to maintain high levels of CD122 on CD8 memory T cells [24]. In addition, genes encoding for epigenetic regulators of transcriptional programs have been attributed important, but distinct functions in Th2 memory cells. First, using a knock-out model it was demonstrated that the polycomb gene *Bmi1* is critical for the survival of CD4 memory T cells through repression of the pro-apoptotic gene *noxa* [25]. Furthermore, the trithorax gene *MLL* was shown to provide activating histone modifications on the *GATA3* and Th2 cytokine loci, which are required for Th2 memory function [26], [27]. Interestingly, both *MLL* and *Bmi1* are also critical for the maintenance of hematopoietic stem cells (HSC) [28]–[30]. Actually, memory T cells and HSCs have several features in common, such as longevity, the ability to self-renew at a very low rate normally followed by re-entering quiescence and the potential to proliferate and differentiate upon cytokine or antigen receptor signalling. Moreover, an overlap was observed in gene expression patterns between memory T, B cells and long-term HSCs [31], supporting the fact that identical molecular pathways might be involved in self-renewal of HSCs and memory T cells.

*Hoxb4* is another well known critical regulator of HSCs and belongs to the family of homeobox (*Hox*) genes, which are transcription factors initially found to determine cell fate in the embryo [32]. The expression of *Hox* genes is epigenetically regulated by the antagonistic actions of polycomb and thrithorax genes [33]. *Hoxb4* is expressed in HSCs, but knock-out mouse models for *Hoxb4* showed that *Hoxb4* is not essential for their generation [34], [35]. However, retroviral mediated overexpression of *Hoxb4* in bone marrow (BM) cells resulted in the expansion of HSCs through promotion of self-renewal divisions without development of overt leukemia in BM chimeric mice [36], [37]. Interestingly, like memory T cells HSCs do not persist in the absence of *Bmi1* [28], [29]. However, *Hoxb4* overexpression could not rescue the long-term maintenance of HSCs deficient for *Bmi1*, despite the triggering of self-renewal of *Bmi1*−/− HSCs [38]. Thus while *Hoxb4* has a function in the execution of the self-renewal division, *Bmi1* provides the HSCs their sustainability. With respect to the resemblances between HSCs and memory T cells, it is likely that these distinct functions might also apply to memory T cells. In this study we set out to evaluate whether *Hoxb4* overexpression would lead to an enhanced self-renewal activity of CD4 MP T cells. Using *Hoxb4* lymphoid specific transgenic mice, we investigated the acute homeostatic proliferation of *Hoxb4* CD4 MP T cells and wild type (wt) MP T cells following transfer in competition into lymphopenic mice. Surprisingly, *Hoxb4* did not provide CD4 MP T cells with an advantage in repopulating the empty T cell niche. In fact the overall contribution of *Hoxb4* cells was significantly lower, but remained rather stable after two additional rounds of homeostatic proliferation. Intriguingly, *Hoxb4* MP T cells consistently comprised a significant larger population of cells expressing surface markers CD62L and Ly6C, indicating a central memory T cell phenotype.

## Materials and Methods

### Ethics Statement

All animal experiments have been performed in accordance with the guidelines of the Canadian Council on Animal Care and have been approved by the Hospital Maisonneuve-Rosemont animal protection committee (protocol number 2012–20).

### Mice

**Figure 1:**
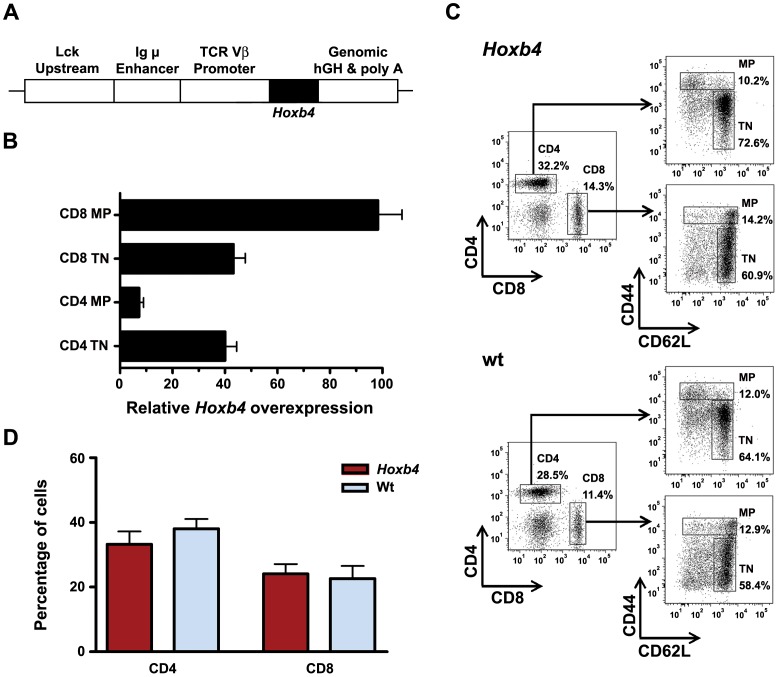
Analysis of T cell populations in *Hoxb4* transgenic mice. (**A**) Scheme of the transgenic construct with lymphoid promoter and enhancer elements from the TCR Vβ, lck and immunoglobulin-μ genes. (**B**) Fold overexpression of *Hoxb4* transgene mRNA in naïve and MP T cells compared to wt. (**C**) Representative FACS profiles of CD4 and CD8 naïve (CD44^lo^/CD62L^hi^) and MP (CD44^hi^) T cell populations in spleen of three months old *Hoxb4* transgenic and wt mice. (**D**) Average percentage of CD4 and CD8 T cells in lymph nodes of *Hoxb4* transgenic (n = 7) and wt mice (n = 6). No significant differences were observed. P>0.05, 2-tailed Student ttest. TN = naïve T cells, MP = memory phenotype T cells, wt = wild type.

*Hoxb4* transgenic mice were generated using the pLIT3 vector (Fig. 1A) and have been described by us before [39]. PCR for the *hGH* gene was performed on genomic tail DNA to identify transgenic mice. Lymphopenic CD3ε^−/−^ recipient mice for transplantation assays have been originally generated by Malissen et al. [40]. Wt mice C57BL/6 (CD45.2) and B6.SJL (CD45.1) were purchased from Jackson Laboratories (Bar Harbor, ME, USA). *Hoxb4* transgenic mice (CD45.2) were bred to B6.SJL mice to generate compound CD45.1/2 *Hoxb4* transgenics. Mice were housed at the animal facility of the Maisonneuve-Rosemont Hospital Research Center under specific pathogen free conditions.

### FACS Analysis and Sorting

To analyse memory T cell populations the following antibodies with conjugated fluorochromes were used: CD8α-PerCP, CD62L-Pacific Blue, Ly6C-Alexa647, Sca-1-PE/Cy7 or Pacific Blue (all obtained from BioLegend, San Diego, CA, USA); CD4-APC/Cy7, CD44-PE/Cy7 or -APC, Ly6C-FITC, CD62L-PE-Cy7 (BD Pharmingen, Mississauga, ON, Canada); CD127-biotin and CD62L-eFluor® 605NC (eBioscience, San Diego, CA, USA). To determine the transgenic or wt origin of MP T cell populations in competitive transplantation assays following antibodies against the CD45 alleles were used: CD45.1-Pacific Blue or -FITC (BioLegend) and CD45.2-APC or -V500 (BD Bioscience). Labelled cells were analyzed on a LSR II with an UV laser (BD Bioscience, Missisauga, ON, Canada), using the Diva software. FlowJo software (TreeStar Inc., Ashland, OR, USA) was used to further analyze specific cell populations.

MP T cell populations for transplantations assays or expression analysis were sorted on a FACSAria III sorter (BD Bioscience) using antibodies for CD44-APC (BioLegend) or -PE-Cy7 (BD Bioscience), CD4 -PerCP, CD8α-PerCP, CD25-biotin and NK1.1-biotin, all from BD Bioscience. Biotin conjugated antibodies were detected with Streptavidin-PE (BioLegend). MP T cells were gated on either CD4+ or CD8+ and further defined as CD44^hi^/CD25^−^/NK1.1^−^.

Antibodies used to determine cytokine production include: IFNγ-FITC (clone B27), TNF-α-PE (clone MP6-XT22), IL-2- APC (JES6-5H4) all purchased from BD Bioscience and their isotype controls: FITC Rat IgG1 (Life Technologies, Burlington, ON, Canada), -PE l5 and -APC l28 (Biolegend).

### Competitive Transplantation Assays

CD4 MP T cells were sorted from spleen and lymph nodes (LNs) of 3 to 4 months old *Hoxb4* transgenic (CD45.1/2) and congenic wt (CD45.1) mice. A cell dose of 2×10^5^ cells composed of equal numbers of *Hoxb4* and wt MP T cells were transplanted in sex matched CD3ε^−/−^ mice by injection in the tail vein. For serial transplantation, mice were sacrificed two months post-transplantation and 10^7^ cells derived from the LNs of each donor were transplanted into a secondary CD3ε^−/−^ host. A third transplantation was repeated again after two months.

For evaluation of short-term proliferation under competitive conditions, sorted *Hoxb4* and wt MP T cells were labelled with CellTrace™ Violet (Life Technologies Inc.) according to the manufacturer’s protocol prior to transplantation. Each recipient received a total dose of 7×10^5^ cells. Mice were sacrificed after one week for analysis.

### Cytokine Analysis

The production of cytokines was measured as described previously [41]. Splenocytes were stimulated with PMA/ionomycin (5 µg/ml) for 2 hours at 37°C, followed by 2 hours incubation with 100 µg/ml Brefeldin A (Sigma-Aldrich Co., St. Louis, MO) to block cytokine secretion. After fixation with formaldehyde (2%) followed by permabilization with 0.5% saponine (Sigma-Aldrich) cells were stained with antibodies against cytokines.

### Quantitative Reverse Transcriptase (Q-RT)-PCR

Total RNA was isolated by Trizol®, DNase-I-treated and cDNA was prepared using MMLV-RT according to the manufacturer’s instructions (Invitrogen, Paisley U.K.). Q-RT-PCR was carried out using SYBRGreen® Power mix (Applied Biosystems, Toronto, ON, Canada), using oligonucleotides for *Hoxb4* and Gapdh as designed before [42], [43]. Reactions were carried out in triplicate. CT-values were corrected for Gapdh expression and to the expression in a calibrator comprised of BM, spleen and LN cells (ΔΔCT). The average fold difference over the expression in the calibrator sample was calculated as 2^(−ΔΔCT)^. To compare the expression of *Hoxb4* in transgenic and wt mice, the average fold difference of *Hoxb4* transgenic was divided by those for the wt mice.

## Results

To evaluate whether overexpression of *Hoxb4* modulates the size of the MP T cell population, a lymphoid specific transgenic mouse for *Hoxb4* was analysed. No major abnormalities in the thymic and splenic lymphoid populations of these mice have been reported [39], but the MP T cell populations have not been analysed in these mice before. To validate the expression of the transgene in MP T cell populations, Q-RT-PCR for *Hoxb4* was performed on RNA purified from CD4 and CD8 T cell subpopulations sorted from LN and spleen of adult *Hoxb4* transgenic and wt mice. Both naïve and MP T cell populations of wt mice expressed *Hoxb4*. Expression levels for *Hoxb4* were markedly increased in transgenic CD4 (7-fold) and CD8 (90-fold) MP T cells (Fig. 1B).

### Memory Phenotype T cell Populations in Young and Aged *Hoxb4* Transgenic Mice

**Figure 2:**
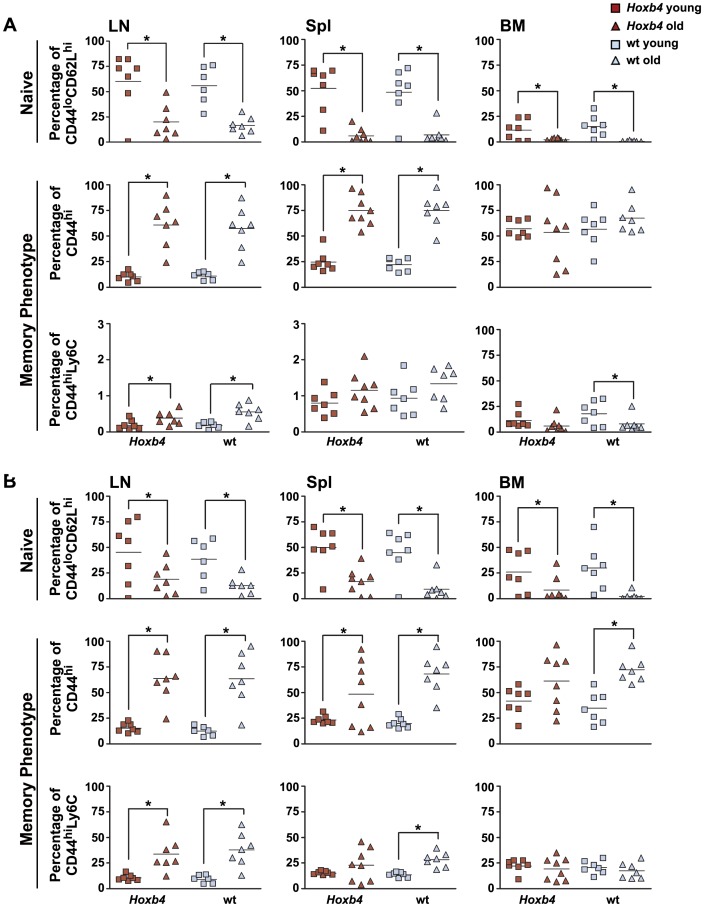
Change of naïve and MP T cell populations in *Hoxb4* transgenic and wt mice with age. Scatter plots showing the percentage of (**A**) CD4 and (**B**) CD8 T cells that are naïve (CD44^lo^/CD62L^hi^), MP (CD44^hi^) or are a subpopulation of MP T cells (CD44^hi^/Ly6C^hi^) for LN, Spl and BM derived from individual *Hoxb4* transgenic and wt (n = 6–8) age matched mice. Young mice are between 3–4 months and old mice are all older than 28 months. Naïve and MP populations change significantly with age, but not between *Hoxb4* and wt mice. *P<0.05, 2-tailed Student ttest. MP = memory phenotype, Wt = wild type, LN = lymph node, Spl = spleen and BM = bone marrow.

FACS analysis showed that CD4 and CD8 T cell populations in BM, spleen and LNs were comparable in adult 2–3 months old *Hoxb4* transgenic and wt mice (Fig. 1C and D; Table 1). Furthermore, naïve T cell (CD44^lo^/CD62L^hi^) and MP T cell populations of the CD4 (CD44^hi^) or CD8 (CD44^hi^ or CD44^hi^/Ly6C^hi^) fractions in these lymphoid organs were not significantly different between *Hoxb4* and wt mice (Fig. 2; Table 1), indicating that intrinsic and extrinsic regulatory mechanisms determining the number of MP T cells are intact in *Hoxb4* transgenic mice.

**Table 1:** Percentage of T cell populations in hematopoietic organs of young adult mice.

Hoxb4			Gated on CD4 or CD8 cells				
	n	Total	CD44^lo^CD62L^hi^	CD44^hi^	CD44^hi^CD62L^hi^	CD44^hi^CD62L^lo^	CD44^hi^Ly6C^+^
***CD4 T cells***							
LN	5	36.99±4.47	71.49±13.87	9.73±4.69	2.18±0.80	6.06±2.83	0.14±0.05
Spleen	7	21.68±8.33	59.22±14.66	24.56±10.32	4.29±0.67	20.59±11.18	0.80±0.37
BM	7	1.86±1.05	11.51±9.97	57.19±8.37	6.65±4.71	50.54±6.90	11.37±7.92
***CD8 T cells***							
LN	5	24.10±8.05	52.73±25.58	14.83±4.75	9.64±4.56	4.55±3.87	10.47±3.07
Spleen	7	10.56±4.93	56.91±9.64	23.24±4.18	16.38±2.88	7.46±4.56	15.06±1.98
BM	7	1.99±1.34	25.94±19.95	41.68±13.84	14.23±9.19	27.44±18.25	22.16±6.35
**Wild Type**			**Gated on CD4 or CD8 cells**				
	**n**	**Total**	**CD44^lo^CD62L^hi^**	**CD44^hi^**	**CD44^hi^CD62L^hi^**	**CD44^hi^CD62L^lo^**	**CD44^hi^Ly6C^+^**
***CD4 T cells***							
LN	6	38.05±7.45	55.80±19.07	10.93±3.90	2.23±0.57	8.69±3.98	0.17±0.09
Spleen	7	20.50±5.57	56.07±12.47	22.17±6.24	4.56±1.44	18.99±5.19	1.02±0.48
BM	7	1.68±0.80	15.30±10.15	56.68±17.49	6.56±4.62	50.13±14.97	17.89±11.75
***CD8 T cells***							
LN	6	22.60±9.60	38.45±20.26	12.36±4.58	6.03±2.95	6.33±5.14	9.17±3.89
Spleen	7	9.36±4.31	52.06±10.42	19.90±4.83	12.72±2.89	7.52±3.23	13.53±2.64
BM	7	1.75±1.65	30.01±21.76	34.78±15.10	12.41±6.61	22.37±12.93	20.60±6.32

Note that no significant differences were observed between T cell populations of *Hoxb4* and wild type mice. 1-tailed Student ttest, comparing *Hoxb4* vs. wild type mice. LN = Lymph node; BM = bone marrow.

It has been well documented that T cell populations decline with age as result of an involuted thymus decreasing production of new naïve T cells [44]. The memory T cell population is relatively stable and might even increase due to accumulation of newly generated memory T cells as result of lifelong exposure to antigens. To evaluate whether changes in proportions of naïve and MP cells with age also occur in T cells overexpressing *Hoxb4*, lymphoid populations of old mice (>15 months) were analyzed and compared to wt mice. First, analysis of wt mice showed that in aged mice the overall percentage of CD4 cells was significantly reduced in LN compared to young mice (Fig. S1A; Table 1 and 2). On the contrary, an increase in CD4 and CD8 T cells was found in the BM of wt mice. As expected naïve CD4 and CD8 T cell subpopulations in aged wt mice were dramatically reduced in LN, spleen and BM (Fig. 2 and Table 1 and 2), while the CD44^hi^ memory populations and their subpopulations CD44^hi^/Ly6C^hi^ were increased, except in the BM for CD4. In *Hoxb4* transgenic mice the decrease in both CD4 and CD8 T cells with age was more pronounced than in wt mice, reaching significance in both LN and spleen (Fig. S1). Similarly as in wt mice, the proportions of naïve CD4 and CD8 subpopulations in old *Hoxb4* transgenic mice were decreased compared to young mice and those of CD44^hi^ memory phenotype T cells increased (Fig. 2; Table 1 and 2). However, the cellularity in hematopoietic organs tended to be reduced in old *Hoxb4* mice (Table S1). This had as consequence that the numbers of CD44^hi^ MP cells were not significantly increased in these mice. Together, these data show that *Hoxb4* does not affect T cell homeostasis in young mice that are in steady-state hematopoiesis.

**Table 2:** Percentage of T cell populations in hematopoietic organs of aged adult mice.

*Hoxb4*			Gated on CD4 or CD8 cells				
	n	Total	CD44^lo^CD62L^hi^	CD44^hi^	CD44^hi^CD62L^hi^	CD44^hi^CD62L^lo^	CD44^hi^Ly6C^+^
***CD4 T cells***							
LN	5	20.44±5.41	19.98±16.36	60.76±21.87	4.39±1.85	56.37±21.20	0.38±0.19
Spleen	7	* **11.43±3.09**	6.08±7.47	73.77±15.76	3.05±1.85	71.76±15.40	1.16±0.35
BM	7	* **2.91±1.60**	* **2.28±1.61**	53.03±34.94	1.32±0.86	52.22±31.56	6.06±6.97
***CD8 T cells***							
LN	5	13.78±7.82	19.07±15.09	63.75±22.69	30.64±14.72	33.11±16.19	33.71±16.88
Spleen	7	* **4.68±1.96**	16.02±16.12	56.93±32.64	17.09±8.75	42.17±24.90	28.65±14.06
BM	7	* **1.51±0.98**	8.45±12.25	63.91±27.87	10.23±7.45	50.98±21.90	19.32±10.93
**Wild Type**			**Gated on CD4 or CD8 cells**				
	**n**	**Total**	**CD44^lo^CD62L^hi^**	**CD44^hi^**	**CD44^hi^CD62L^hi^**	**CD44^hi^CD62L^lo^**	**CD44^hi^Ly6C^+^**
***CD4 T cells***							
LN	6	22.49±7.55	16.74±7.90	57.35±20.85	5.64±3.04	51.72±17.96	0.56±0.24
Spleen	7	18.12±5.57	6.94±9.65	74.77±16.12	2.98±1.07	71.79±15.60	1.33±0.47
BM	7	5.55±1.50	0.76±0.44	67.47±14.67	2.01±1.22	65.47±14.44	8.14±7.67
***CD8 T cells***							
LN	6	17.38±10.71	12.88±8.31	63.47±26.18	37.33±26.09	26.15±8.15	37.87±16.45
Spleen	7	9.26±5.48	9.25±10.95	60.09±32.16	25.22±11.54	42.91±10.96	28.36±7.18
BM	7	3.60±2.06	2.41±3.74	72.30±12.48	11.76±9.39	60.54±12.22	17.50±7.77

Note the decrease in total T cells in bone marrow and spleen of *Hoxb4* mice;

* P<0.05;

1-tailed student ttest, comparing *Hoxb4* vs. wild type mice. LN = Lymph node; BM = bone marrow.

### Short-term Competitive Homeostatic Proliferation of CD4 Memory T cells

**Figure 3:**
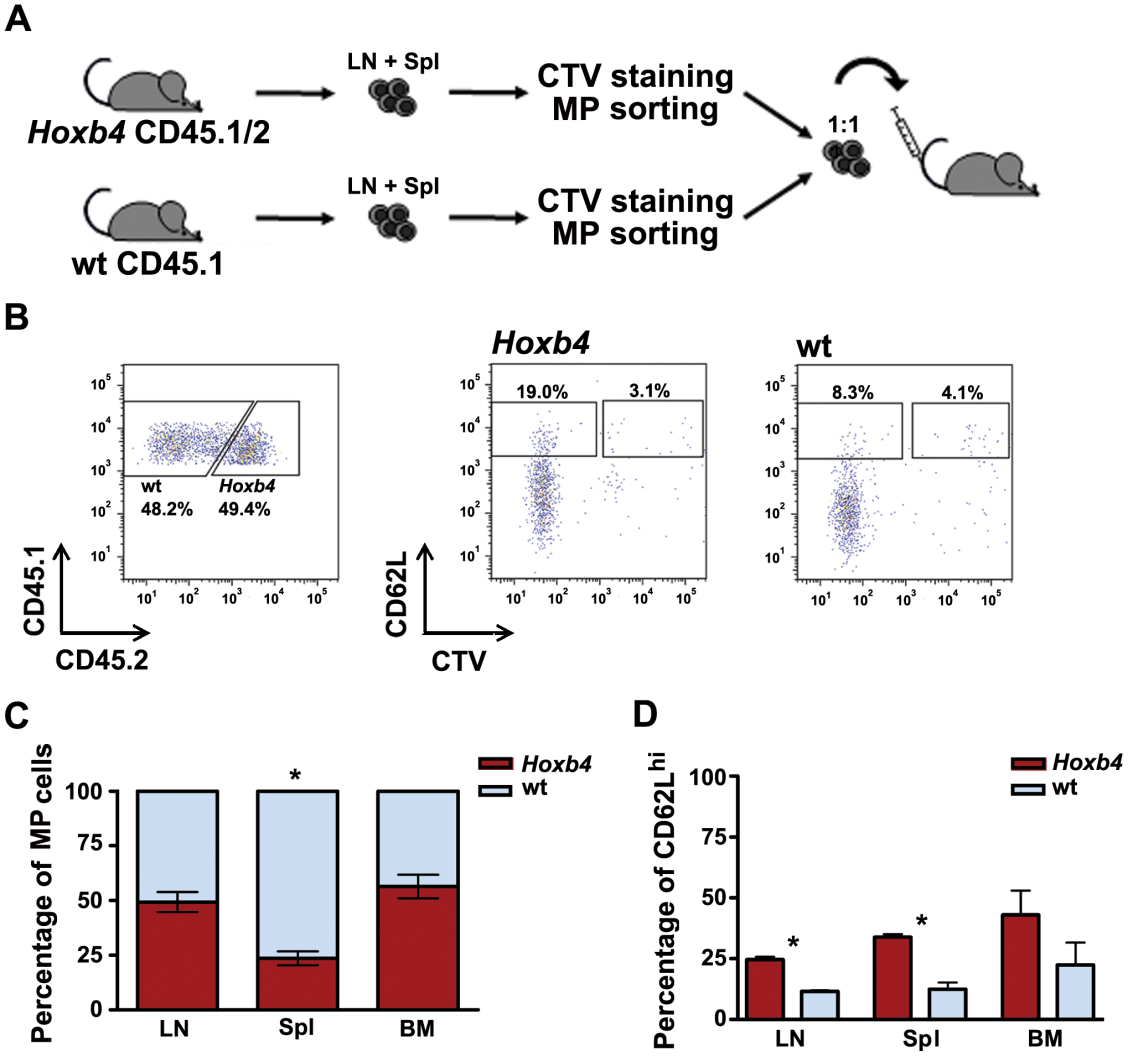
Competitive short-term homeostatic proliferations (7 days) of *Hoxb4* transgenic and wt CD4 MP T cells. (**A**) Scheme of the experimental approach. CD4 MP T cells are sorted from CellTrace™ Violet (CTV) labelled cells isolated from LN and Spl of *Hoxb4* (CD45.1/2) and congenic wt (CD45.1) mice. Cells of both genotypes are transplanted in a 1∶1 ratio in CD3ε^−/−^ (CD45.2) mice. (**B**) FACS profiles showing *Hoxb4* and wt fractions to donor derived CD4 MP T population (CD45.1) in LN (left panel). Representative FACS profiles for CD62L and CTV on *Hoxb4* and wt populations. Loss of CTV tracer indicates that most cells are dividing rapidly (right panels). (**C**) Average contribution (%) of *Hoxb4* and wt cells to donor derived MP T cells in LN, Spl and BM (n = 3). (**D**) Percentage of CD62L^hi^ MP T cells in *Hoxb4* and wt population found in lymphoid organs. *P<0.05; paired 2-tailed Student ttest. Wt = wild type, MP = memory phenotype, LN = lymph node, Spl = spleen and BM = bone marrow.

Adoptive transfer of T cells into a lymphopenic host results in a rapid proliferation of these cells to fill the empty niche. To test whether *Hoxb4* CD4 MP T cells have an advantage over wt cells in the initial proliferation phase following transfer, CD4 MP cells (CD44^hi^/CD25^−/^NK1.1^−^) were sorted from both *Hoxb4* transgenic (CD45.1/2) and wt congenic mice (CD45.1), stained with CellTrace™ Violet (CTV) and transplanted in a 1∶1 ratio into CD3ε^−/−^ mice lacking T cells (Fig. 3A). One week post transplantation, the mice were sacrificed and analysed for the presence of donor cells by fluorescence activated cell sorting (FACS). Distinct populations of donor cells were detected in all organs representing 0.20% of the total cell population in both LNs and spleen and 0.03% in BM (data not shown). *Hoxb4* and wt CD4 MP T cells contributed equally to LNs and BM of the recipient mice, but in the spleen the wt cells dominated over *Hoxb4* MP T cells (Fig. 3B left panel and 3C). Both *Hoxb4* and wt MP T cells underwent several divisions during seven days, because the majority of cells were negative for CTV (Fig. 3B).

Interestingly, the proportions of cells expressing the surface marker CD62L, which is characteristic for central memory T cells, were higher in *Hoxb4* CD4 MP T cells (Fig. 3D).

Together these data show that both *Hoxb4* and wt CD4 MP T cells have divided rapidly to occupy the niche in T cell deficient mice with fluctuations in the different organs. Moreover, overexpression of *Hoxb4* promoted the enrichment of CD62L positive cells following lymphopenia induced proliferation.

### Medium-term Competitive Repopulation in a Lymphopenic Host

**Figure 4:**
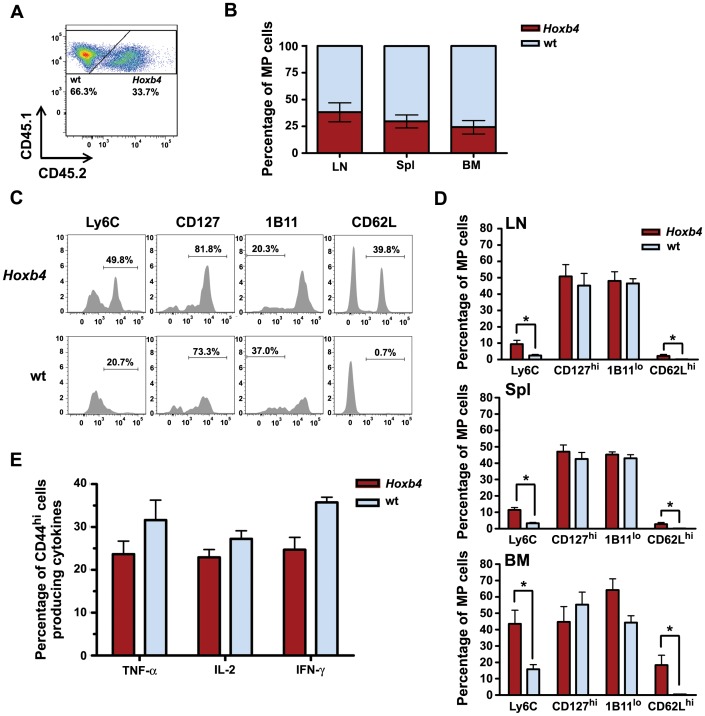
Medium-term competitive homeostatic proliferations (60 days) of *Hoxb4* transgenic and wt CD4 MP T cells. (**A**) FACS profile showing fractions of *Hoxb4* and wt cells to donor derived MP T population in lymph node (LN). (**B**) Stacked bar graphs indicating the average contributions of *Hoxb4* and wt cells in LN, Spl and BM measured in three independent experiments; n = 9. (**C**) FACS profiles for the expression of typical memory T cell surface markers on *Hoxb4* and wt MP T cells in the BM. (**D**) Average subpopulations of *Hoxb4* and wt MP T cells expressing the indicated surface markers in LN (upper panel), Spl (middle panel) and BM (lower panel). (**E**) Percentage of *Hoxb4* and wt MP T cells (gated on CD44^hi^) positive for indicated cytokines (n = 3–6). *P<0.05, 2-tailed Student ttest. MP = memory phenotype, wt = wild type, LN = lymph node, Spl = spleen and BM = bone marrow, TNF = tumor necrosis factor; IL-2 = interleukine-2; IFN = interferon.

To evaluate whether *Hoxb4* CD4 MP T cells would dominate over the wt MP T cells with time, equal numbers of *Hoxb4* (CD45.1/.2) and wt (CD45.1) CD4 MP T cells were transplanted in CD3ε^−/−^ mice and sacrificed after two months. No signs of disease were observed in these mice. At this time point the transferred MP T cells occupied at average 6.6±3.1% of the cells in LN, 4.5±1.7% in the spleen and 0.7±0.8% in the BM of recipient mice (Table 3). This is equivalent with a total of ∼6.4×10^6^ donor cells in these organs, which implies that MP T cells have expanded at least 31-fold over the number of injected cells (data not shown). The average contribution of wt CD4 MP T cells to the repopulation of the BM and spleen in nine individual recipients transplanted in three independent experiments was 2- to 3-fold higher in the spleen and BM than that of *Hoxb4* CD4 MP cells (Table 3 and Fig. 4A and B). However, the percentage of the *Hoxb4* and wt CD4 MP T cells in the LNs of host mice was very variable and not significantly different (Fig. 4B and Table 3). Total *Hoxb4* and wt CD4 MP T cells in the combined organs were calculated and showed a net expansion of 20-fold and 43-fold for *Hoxb4* and wt cells, respectively (Fig. S2A). This resulted in a net 2-fold higher contribution of wt CD4 MP T cells over *Hoxb4*. Both *Hoxb4* and wt CD4 MP T cell populations were further characterized for the presence of specific surface markers such as CD62L, Ly6C, CD127 and 1B11. FACS analysis showed that the percentage of Ly6C^+^ cells was 3- to 4-fold higher in the *Hoxb4* population compared to wt in all organs analysed (Fig. 4C and D). Moreover, in contrast to a negligible proportion of wt CD4 memory T cells that express CD62L (<1.0% in all organs), a distinct proportion of *Hoxb4* cells expressed this marker in BM (13.9±7.1%), spleen (3.4±1.3%) and LN (3.3±1.2%), indicating that *Hoxb4* favours central memory characteristics. All CD62L^+^ cells carried also the Ly6C marker (data not shown). The percentage of cells expressing CD127, CD43 and CD44 was not different for both genotypes. Since central memory T cells are considered the long-term memory T cells, we analysed if *Hoxb4* overexpression act more specifically on the expansion of CD4 central MP T cells. The total numbers of CD62L^+^ cells in BM, spleen and LNs of the *Hoxb4* population ranged from 7000 to 41000 (mean 22821±13083) and of the wt population from 160 to 5000 (mean 2030±1912; Fig. S2B). The percentage of CD62L^+^ CD4 MP T cells in the initial graft were considered equal based on data in Table 1 and estimated at 20000 cells. After competitive homeostatic proliferation the number CD62L^+^ cells had increased in the *Hoxb4* population in four out of six mice. In contrast the CD62L^+^ MP T cells were decreased in the wt population for all six mice. In addition to the molecular make-up of the MP T cells, the functional response after stimulation with PMA was measured. The proportion of *Hoxb4* MP T cells that produced TNF-α and IL-2 was somewhat, but not significantly, reduced. However, a modest, but significant decrease in the number of *Hoxb4* cells producing IFN-γ was observed (Fig. 4E). Thus these data show that in the majority of mice *Hoxb4* CD4 MP T cells were less competitive, but functional. Furthermore, the subset of more central memory like CD62L positive cells was larger in the *Hoxb4* MP T cells and in the presence of *Hoxb4* this population was actually expanded in some mice over the initial transplanted cells.

**Table 3:** Total donor cells contribution to hematopoietic organs and the proportion of *Hoxb4* versus wild type cells.

	LN	Spleen	BM
***primary hosts, n = 9***	(%)	(%)	(%)
** Total**	6.6±3.1	4.5±1.7	0.7±0.8
*** Hoxb4***	38.2±25.5	29.6±18.3	24.2±18.3
** wt**	61.6±25.5	70.2±18.4	75.5±19.2
***secondary hosts, n = 6***			
** Total**	8.7±3.8	3.8±1.6	0.2±0.1
*** Hoxb4***	32.4±15.6	33.4±8.4	34.4±17.2
** wt**	68.7±17.2	66.4±8.1	65.6±17.2
***tertiary hosts, n = 3***			
** Total**	4.4±2.0	1.0±0.7	0.2±0.1
*** Hoxb4***	29.4±4.1	22.5±17.5	24.9±11.0
** wt**	39.9±24.6	35.0±31.9	40.5±28.7

LN = Lymph node; BM = bone marrow, wt = wild type.

### Evaluation of Long-term Competitive Repopulation in Lymphopenic Hosts by Serial Transplantation

**Figure 5:**
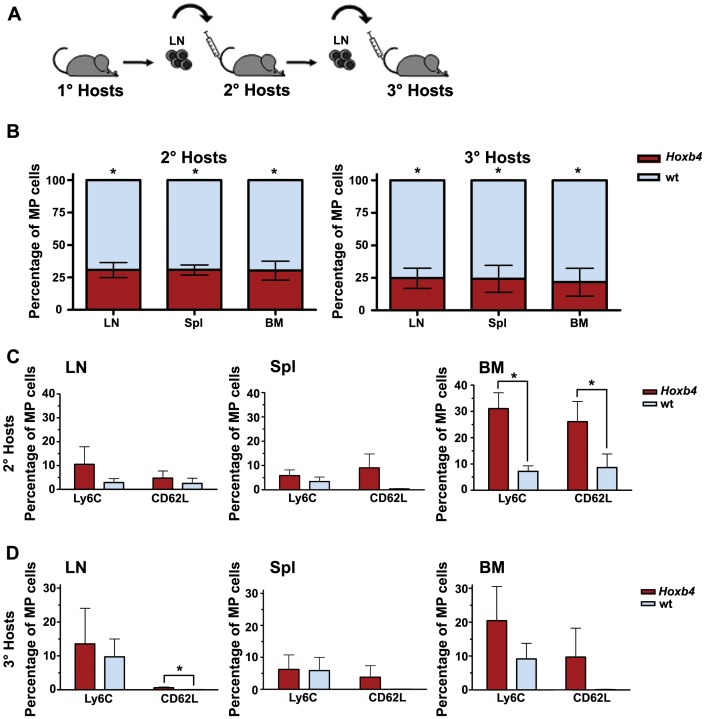
Long-term competitive homeostatic proliferations (180 days) of *Hoxb4* transgenic and wt CD4 cells. (**A**) Scheme of serial transplantations. 10×10^6^ cells of the LNs of primary hosts that received a transplant composed of equal doses of *Hoxb4* and wt MP T cells were serially transplanted into secondary and tertiary hosts with a 60 days interval. (**B**) Compilation of *Hoxb4* and wt fractions of donor derived cells in LN, Spl and BM of secondary (n = 6) and tertiary hosts (n = 4) from two independent experiments. (**C**) Bar graphs showing the average percentage of cells positive for CD62L and Ly6C within the *Hoxb4* or wt memory T populations. *P<0.05, 2-tailed Student ttest. Wt = wild type, MP = memory phenotype, LN = lymph node, Spl = spleen and BM = bone marrow.

Central memory T cells have a more extended live span than effector memory T cells and are considered the long-term memory cells. The *Hoxb4* CD4 MP T population comprised a higher number of CD62L^+^ memory T cells than the wt after one round of homeostatic proliferation. To evaluate whether the contributions of *Hoxb4* and wt MP T cells changed in favour of *Hoxb4* after several rounds of homeostatic proliferation, 10^7^ cells of total LN isolated from six primary hosts were serially transplanted (Fig. 5A), which comprises between 0.5 and 1.0×10^6^ donor CD4 MP T cells. The percentage of transferred MP T cells detected in the hematopoietic organs of the secondary hosts after two months of expansion was not significantly different than in primary hosts (Table 3). Evaluation of the proportions of *Hoxb4* and wt MP T cells by FACS showed that the average contribution of *Hoxb4* MP T cells was significantly lower than that of wt in all organs (Fig. 5B and Table 3). However, only in one out of six mice the contribution of *Hoxb4* was higher than that of wt MP T cells in LN and BM. These ratios of *Hoxb4* vs. wt MP T cells were maintained upon transfer and expansion in a tertiary host. Interestingly, the proportion of CD62L^+^ and Ly6C^+^ cells remained consistently higher in the *Hoxb4* CD4 MP T cell population following serial transfer (Fig. 5C and D). This translated in an actual higher number of *Hoxb4* than wt CD62L^+^ cells in five of six secondary hosts (data not shown). Cytokine analysis following stimulation *in vitro* showed that even after several rounds of expansion *Hoxb4* MP T cells were functionally intact (Fig. S3). Thus despite an increased population of CD62L^+^ MP T cells, the contribution of *Hoxb4* CD4 MP T cells did not change after three rounds of expansion.

## Discussion

Memory T cells are triggered to proliferate following the transplantation into a lymphopenic host through increased availability of IL-7 and likely IL-15 [3], [6]. In this study we investigated whether *Hoxb4*, known for its potential to expand HSCs, also could increase the pool of CD4 MP T cells. Competitive transplantation of *Hoxb4* transgenic and wt CD4 MP T cells showed that *Hoxb4* overexpression did not provide the total MP T cell population with a proliferative advantage. On the contrary, the overall population of *Hoxb4* MP T cells were less competitive in expansion to occupy the empty niche of CD3ε^−/−^ mice than wt CD4 MP T cells. The disadvantage became already apparent in the spleen after 7 days of homeostatic proliferation, despite the fact that most *Hoxb4* CD4 MP T cells had undergone a rapid proliferation (Fig. 3B), and was even more pronounced after 2 months. Two additional rounds of homeostatic proliferation did not further change the established ratios between *Hoxb4* and wt MP T cells. We cannot completely exclude that *Hoxb4* CD4 MP T cells were lost by apoptosis, but we think that this is not very likely, because no differences in apoptosis within the MP T cell populations were observed in *Hoxb4* transgenic and wt mice (data not shown).

A major observation is the enrichment of CD62L and Ly6C subpopulations in the presence of *Hoxb4* overexpression. The CD62L molecule is a classical indicator for central memory T cells [45]. Although Ly6C has been recognized as a marker for CD8 central memory T cells, this is less clear for CD4 memory T cells. Tokoyoda et al. demonstrated that antigen specific CD4 memory T cells preferentially reside in the BM and express high levels of Ly6C, but these cells did not express central memory molecules CD62L or CCR7 [46]. The Ly6C expression on CD4 memory T cells still remains controversial as a more recent study by Marshall et al. demonstrates that Ly6C^lo^ effector cells persisted better during the contraction phase than Ly6C^hi^ cells and thus are more prone to develop into memory T cells [47]. In the same study they show that the transcriptional profile of Ly6C^lo^ effector T cells resembled those of memory T cells. Interestingly, high expression of Ly6C was observed once the effector T cells were converted into memory T cells. It is thus not clear whether Ly6C^+^ cells could be considered central memory cells. We found that CD62L expressing cells expressed also Ly6C, but in addition Ly6C^+^/CD62L^−^ cells were present. The expression of Ly6C was mostly low to intermediate, while only a small fraction of cells were Ly6C^hi^ and thus does not contradicts observations mentioned by Marshall et al. It is of interest to note that we observed highest proportions of Ly6C and CD62L cells in the BM, which has been allocated as the principal niche for long-term CD4 memory T cells [46]. The actual increase of the CD62L population by *Hoxb4* found in several mice could be achieved by promotion of self-renewal divisions as has been suggested for *Hoxb4* [36]. Thus our data suggest that *Hoxb4* might indeed favour self-renewal of CD4 MP T cells, but only the stem cell like CD62L^+^ central memory T cells. Alternatively, *Hoxb4* could activate CD62L or Ly6C expression, however, no binding sites for Hoxb4 or its cofactor Pbx are predicted on the promoter sequences of either gene according to the DECipherment Of DNA Elements (DECODE) database, which compiles predicted binding sites for over 200 transcription factors, suggesting no direct activation. Indirect activation of CD62L by Hoxb4 or through binding to more distant enhancer regions cannot be excluded, but is unlikely as populations of CD44^+^/CD62L^+^ in transgenic *Hoxb4* mice are not enhanced compared to wild type.

In our experimental design, memory T cells were purified from non-immunized mice and are considered MP CD4 T cells that have been generated in the absence of antigen during homeostatic proliferation. It is well known that MP CD4 T cells are a heterogenous population of cells [3]. A subset of these cells has been shown to divide more rapidly than antigen specific memory T cells. This proliferation appeared to be independent of homeostatic cytokines, but these MP CD4 cells do require contact with MHCII, possibly loaded with foreign antigens, for their homeostatic proliferation. It has been reported that these fast dividing CD4 MP T cells have some properties of effector cells [3] and it is thus possible that *Hoxb4* is not favouring the expansion of this subpopulation. A potential reason for the reduced proliferation of the *Hoxb4* CD4 MP T cells might be an effect of *Hoxb4* on thymic differentiation and the TCR repertoire. However, we did not observe any anomalies of thymic T cell differentiation as thymic FACS profiles showed a normal distribution of thymic cell subsets [39], indicating that *Hoxb4* does not interfere with T cell development and thus making this possibility less likely. Furthermore, analysis of the TCR Vβ usage by peripheral T cells did not show any difference in repertoire between wt and *Hoxb4* transgenic mice (data not shown), indicating that a change in TCR repertoire is probably not the reason for the reduced homeostatic expansion of *Hoxb4* CD4 MP T cells in our experiments.

Based on the enrichment of CD4 central MP T cells in the *Hoxb4* population it was expected that *Hoxb4* MP T cell would dominate the repopulation lymphopenic hosts. It is not entirely clear why after three rounds of homeostatic proliferation the contribution of *Hoxb4* CD4 MP T cells did not increase. Recently, the gut has been identified as an important reservoir for CD4 memory T cells [48]. One possibility is that *Hoxb4* CD4 MP T cells preferentially migrated to the gut site and were not included in our analysis. This requires the expression of adhesion molecule, integrin α4β7. Interestingly, the DECODE database mentions binding sites for Hoxa9 and Meis1 in the promoter of the *itga4* and *itgb7* genes coding for integrin α4 and β7, respectively. Although Hoxb4 is not included in their list it is possible that Hoxb4 might target this gene as well, either directly or through complex with Pbx and Meis1. Of note is that *Hoxa9* also has the potential to expand HSCs [49], and another integrin sharing the same alpha chain, α4β1 (VLA-4) is expressed on HSCs, allowing for adhesion to the BM stroma [50]. Thus an increase in α4β7 expression on *Hoxb4* CD4 MP T cells resulting in enhanced homing to the gut cannot be excluded, and could explain a lower contribution of *Hoxb4* CD4 memory T cells to the lymphoid organs.

In addition, it is still plausible that CD4 MP T cells might respond differently to *Hoxb4* than HSCs. It might be that homeostatic proliferation of MP T cells is predominantly governed by cytokine signalling, while intrinsic signalling pathways play a larger role in HSCs.

In conclusion we show that *Hoxb4* favours the maintenance and expansion of CD4 central MP cells following acute homeostatic proliferation, which suggest a more robust preservation of CD4 MP T cells in the long-term.

## Supporting Information

Figure S1 **Analysis of T cell populations in young and old** ***Hoxb4*** **transgenic mice.** Graphs showing the average size of CD4 **(A)** and CD8 **(B)** populations in lymphoid organs of young (2–3 months of age) and old (>15 months of age) *Hoxb4* transgenic (n = 7) and wt (n = 7) age matched mice. *P<0.05, 2-tailed Student ttest. Wt = wild type, LN = Lymph node; BM = bone marrow.
(TIF)

Figure S2 **Absolute CD4 MP T cell numbers following homeostatic proliferation. (A)** Absolute number of *Hoxb4* and wt CD4 MP T cells in lymphoid organs of primary hosts after 2 months of competitive proliferation. The calculations of the absolute numbers are based on 8 LNs, Spl and BM derived from 2 legs. Data are obtained from 9 mice in 3 independent experiments. *P = 0.03; 2-tailed Student ttest. **(B)** Absolute number of *Hoxb4* and wt CD62L positive CD4 MP T cells in primary hosts (n = 6). The numbers of CD62L MP T cells in the initial graft are calculated based on percentage of CD44^hi^/CD62L^+^ population as given in Table 1. Note the expansion of the CD62L population in several mice. *P = 0.01; 2-tailed Student ttest. Wt = wild type, MP = memory phenotype, LN = Lymph node, Spl = spleen, BM = bone marrow.
(TIF)

Figure S3 **Production of cytokines after stimulation with PMA/ionomycin.** Percentage of *Hoxb4* and wt MP T cells (gated on CD44^hi^) in secondary and tertiary hosts positive for indicated cytokines (n = 3–6). Wt = wild type, MP = memory phenotype, TNF = tumor necrosis factor; IL-2 = interleukine-2; IFN = interferon.
(TIF)

Table S1 **Average cell numbers (×106) in hematopoietic organs of** ***Hoxb4*** **and wt mice.**
(DOC)
