# Supplementary figures and images for: Hoxb4 Overexpression in CD4 Memory Phenotype T Cells Increases the Central Memory Population upon Homeostatic Proliferation
Source: PLoS One. 2013 Dec 6;8(12):e81573. doi: 10.1371/journal.pone.0081573 (PMC3855745; doi:10.1371/journal.pone.0081573)

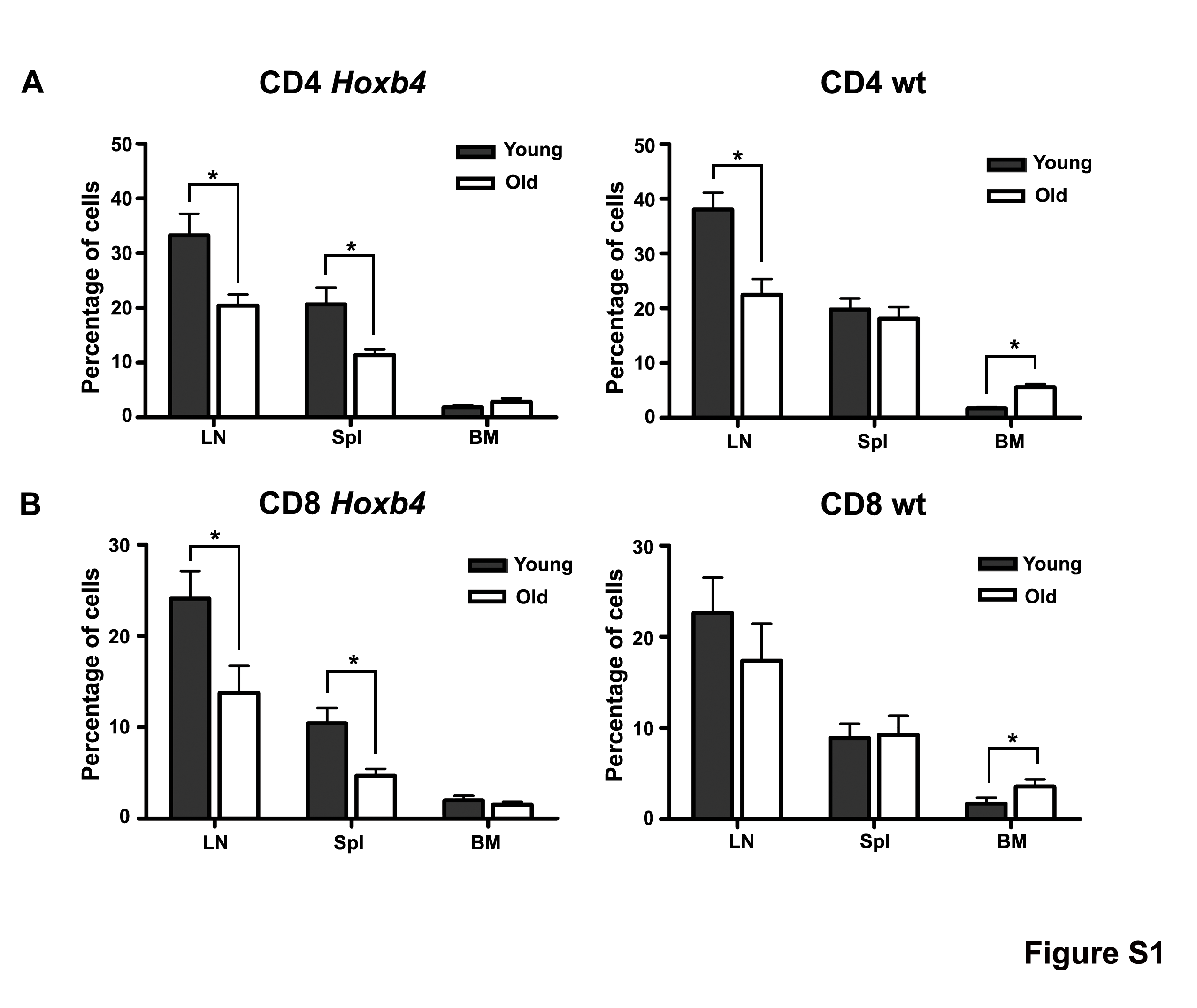

Supplement: Figure S1 — Analysis of T cell populations in young and old Hoxb4 transgenic mice. Graphs showing the average size of CD4 (A) and CD8 (B) populations in lymphoid organs of young (2–3 months of age) and old (>15 months of age) Hoxb4 transgenic (n = 7) and wt (n = 7) age matched mice. *P<0.05, 2-tailed Student ttest. Wt = wild type, LN = Lymph node; BM = bone marrow. (TIF) [file pone.0081573.s001.tif]

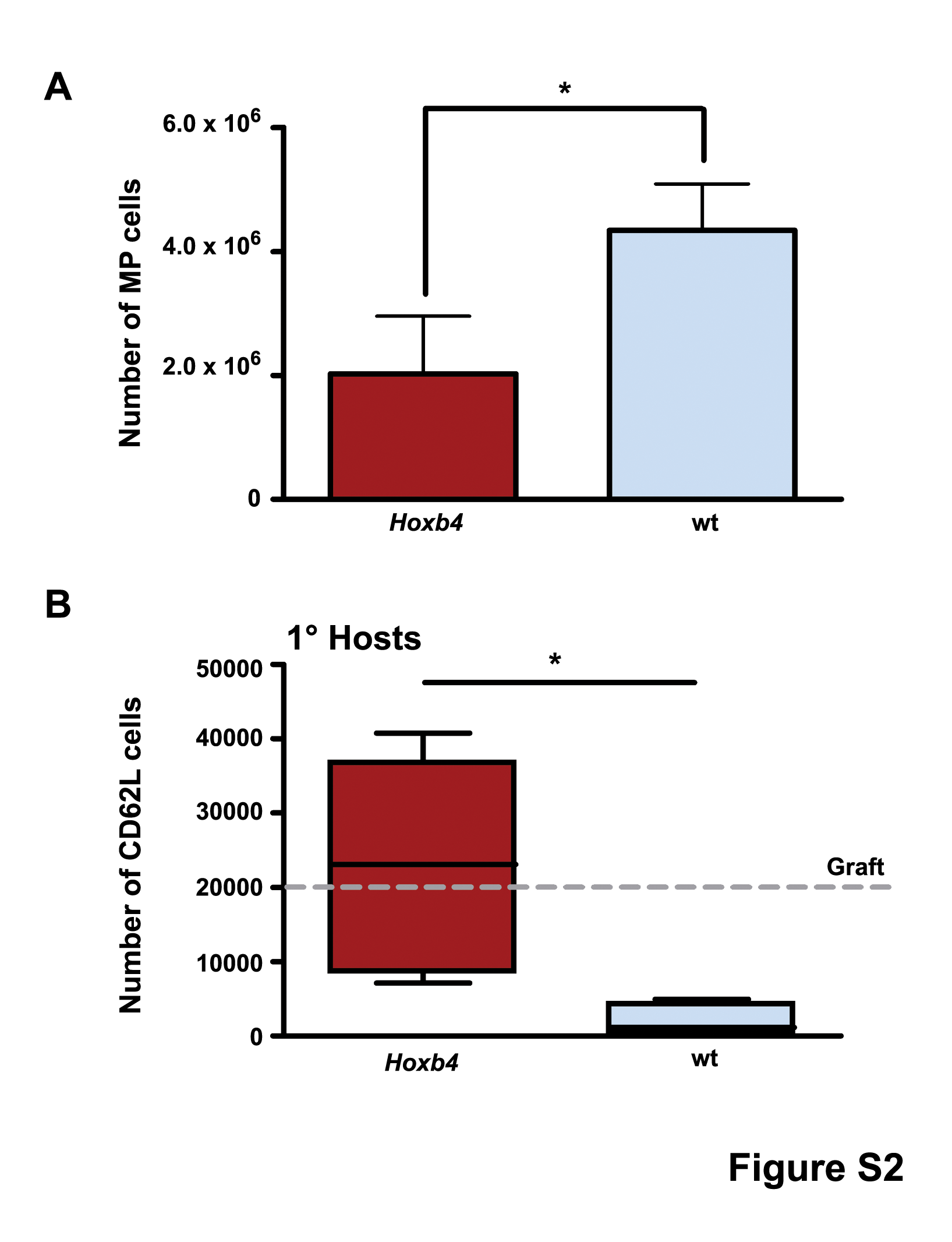

Supplement: Figure S2 — Absolute CD4 MP T cell numbers following homeostatic proliferation. (A) Absolute number of Hoxb4 and wt CD4 MP T cells in lymphoid organs of primary hosts after 2 months of competitive proliferation. The calculations of the absolute numbers are based on 8 LNs, Spl and BM derived from 2 legs. Data are obtained from 9 mice in 3 independent experiments. *P = 0.03; 2-tailed Student ttest. (B) Absolute number of Hoxb4 and wt CD62L positive CD4 MP T cells in primary hosts (n = 6). The numbers of CD62L MP T cells in the initial graft are calculated based on percentage of CD44hi/CD62L+ population as given in Table 1. Note the expansion of the CD62L population in several mice. *P = 0.01; 2-tailed Student ttest. Wt = wild type, MP = memory phenotype, LN = Lymph node, Spl = spleen, BM = bone marrow. (TIF) [file pone.0081573.s002.tif]

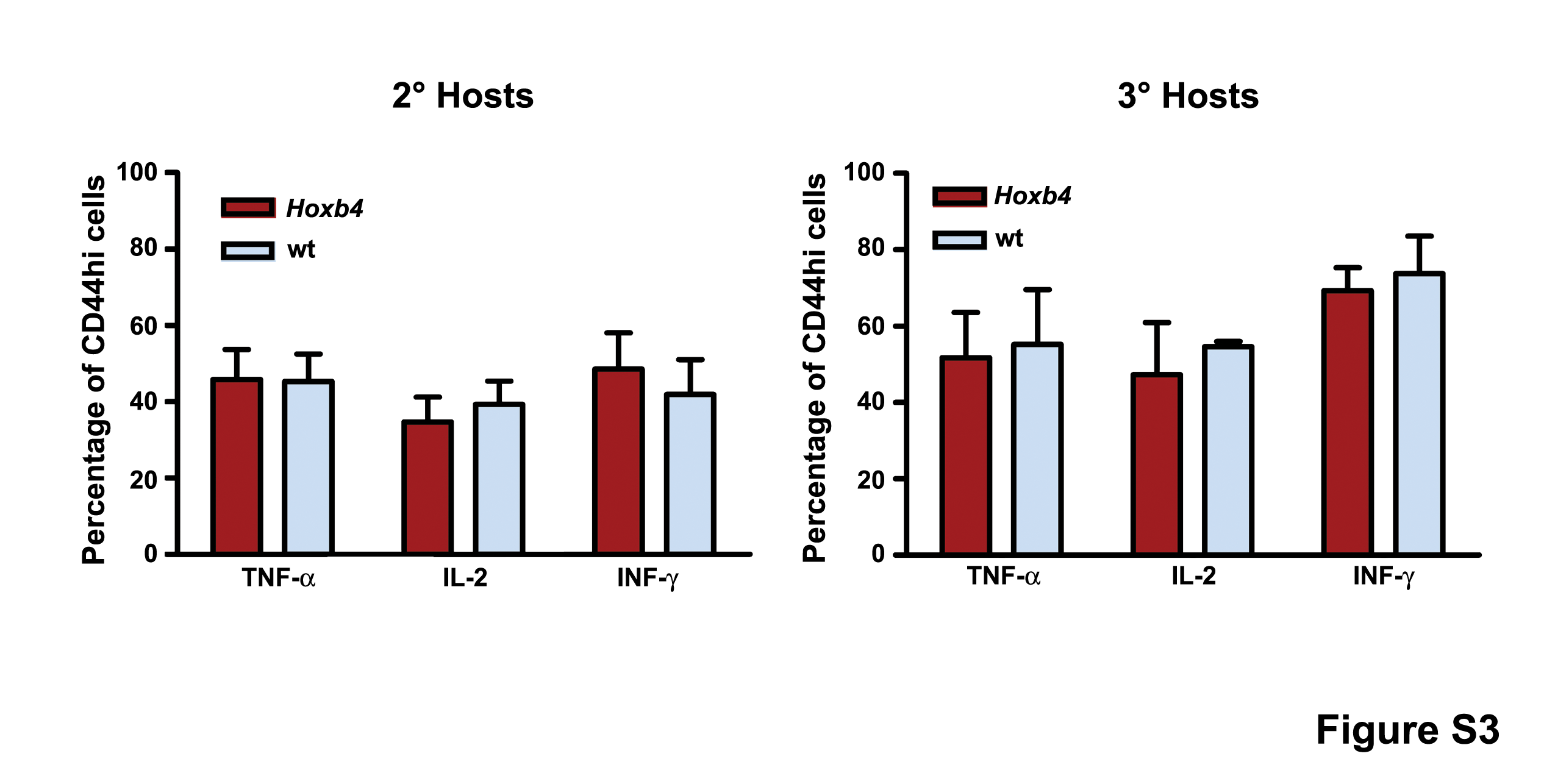

Supplement: Figure S3 — Production of cytokines after stimulation with PMA/ionomycin. Percentage of Hoxb4 and wt MP T cells (gated on CD44hi) in secondary and tertiary hosts positive for indicated cytokines (n = 3–6). Wt = wild type, MP = memory phenotype, TNF = tumor necrosis factor; IL-2 = interleukine-2; IFN = interferon. (TIF) [file pone.0081573.s003.tif]
