# Supplementary material for: Hoxb4 Overexpression in CD4 Memory Phenotype T Cells Increases the Central Memory Population upon Homeostatic Proliferation
Source: PLoS One. 2013 Dec 6;8(12):e81573. doi: 10.1371/journal.pone.0081573 (PMC3855745; doi:10.1371/journal.pone.0081573)
Supplement: Table S1 — Average cell numbers (×106) in hematopoietic organs of Hoxb4 and wt mice. (DOC) [file pone.0081573.s004.doc]

**Table S1 Average cell numbers (x10^6^) in hematopoietic organs of *Hoxb4* and wt mice**

|  | **LN** | **Spleen** | **BM** |
| --- | --- | --- | --- |
| **Young** |  |  |  |
| *Hoxb4* | 34.95 ± 17.94 | 208.08 ± 77.09 | 31.75 ± 11.12 |
| wt | 34.91 ± 14.21 | 156.18 ± 56.26 | 33.37 ± 8.36 |
|  |  |  |  |
| **Old** |  |  |  |
| *Hoxb4* | 22.25 ± 5.54 | 153.47 ± 24.58 | 25.08 ± 5.49 |
| wt | 30.75 ± 10.09 | 192.57 ± 93.61 | 29.30 ± 9.11 |

wt = wild type; LN = lymph node; BM = bone marrow
